# Hepatitis B virus sero-prevalence amongst pregnant women in the Gambia

**DOI:** 10.1186/s12879-019-3883-9

**Published:** 2019-03-15

**Authors:** Mustapha Bittaye, Patrick Idoko, Bissallah Ahmed Ekele, Samuel Amenyi Obed, Ousman Nyan

**Affiliations:** 1 Edward Francis Small Teaching Hospital, Banjul, The Gambia; 2 University of Abuja Teaching Hospital, Gwagwalada, Nigeria; 3 Korle Bu Teaching Hospital, School of Medicine, University Of Ghana, Accra, Ghana; 4 School of Medicine & Allied Health Sciences, University of The Gambia, Brikama, Gambia

**Keywords:** Pregnancy, Hepatitis B infection, Sero-prevalence

## Abstract

**Background:**

Infection with Hepatitis B virus (HBV) is a serious public health problem worldwide, with over 360 million carriers. Sixty million of these are resident in Sub-saharan Africa. Hepatitis B infection is the cause of Hepatocellular carcinoma (HCC), which is the second commonest cause of death from cancers among women in The Gambia. Vertical transmission is the commonest route of spread of Hepatitis B Virus in many endemic areas. The main aim of the study was to determine the sero-prevalence of Hepatitis B surface antigen (HBsAg) among pregnant women attending antenatal clinic at the Edward Francis Small Teaching Hospital, Banjul, The Gambia.

**Methods:**

Four hundred and twenty six pregnant women were recruited from our antenatal clinics and tested for HBsAg. Serum Hepatitis B surface antigen (HBsAg) was tested using commercial rapid diagnostic Elisa kits at the point of care.

**Results:**

A prevalence rate of 9.20% among all pregnant women studied was found. Women who were likely to have been vaccinated had a prevalence rate of 2.30% whiles those unlikely to have been vaccinated had a prevalence of 13.71%. There was a statistically significant difference between those likely to have been vaccinated and those unlikely to have been vaccinated.

**Conclusion:**

The prevalence of hepatitis B infection is very high among pregnant women at EFSTH as in the high endemic zone that is more than 8%. However the prevalence rate is lower than the national average of 15%. The prevalence is of moderate endemicity among the women who likely received vaccination during childhood. More interventions during pregnancy need to be undertaken if more successes are to be registered.

## Background

World Health Organisation (WHO) in its 2009 update has shown that there were globally over 2 billion people estimated to be living with the hepatitis B virus, while around 360 million are chronically infected and at risk of developing life-threatening liver disease [1]. Sixty million of these chronically infected reside in sub-Saharan Africa. Up to a quarter of these chronically infected individuals may die as a result of the infection [1]. It is estimated that more than half a million deaths per year are as a result of hepatitis B virus infection [1].

The Gambia is in an area of high prevalence of hepatitis B infection. The classification of high prevalence is a prevalence of 8% or more [2]. A study done in The Gambia has suggested that HCC is more likely in individuals who perinatally established chronic infection through infectious mothers than in those infected with HBV by horizontal transmission [3]. This paper also showed that interrupting vertical transmission in Sub-Saharan Africa will help reduce the burden of liver disease [3].

Ten to 15 % of all adult male deaths in The Gambia are estimated to be related to hepatocelluar carcinoma (HCC) and chronic liver disease [4]. Hepatitis B infection is the cause of HCC which is the second commonest cause of death from cancers among women in The Gambia [5].

There are many ways the hepatitis B infection gets transmitted from one individual to another. The pattern of transmission depends on the number of chronic carriers in the community. In areas where it is endemic such as Sub-Saharan Africa, transmission is mainly either vertical, from a carrier mother to her newborn, or through close contact between children. Perinatal transmission of HBV occurs mainly during or soon after delivery, through contact of the infant with maternal blood and other body fluids [6]. Breast milk has not been implicated as a major source of vertical transmission but mothers who are carriers are encouraged not to wet-nurse other babies but can breast feed their own children [6]. Mothers who are carriers with the “e” antigen positive have a 90% chance of infecting their new born [7]. It has been shown that children infected before 1 year have a 90% chance of becoming chronic carriers and only 10% of those infected at adulthood end up being chronic carriers [7]. It is thus very relevant that all pregnant women are screened during antenatal care for hepatitis B infection and those positive have their infants treated with a specific hyperimmune globulin, (Hepatitis B Immune Globulin or HBIG and hepatitis B vaccine (HBVc) [8].

WHO has suggested that the main intervention to reduce hepatitis B infection is by increasing the coverage of infant vaccination [9]. Vaccination reduces the risk of developing hepatitis B infection among infants of hepatitis B positive mothers by 3.5 times [9]. It has also shown that critical in the vaccination is when the first vaccine was taken after birth. It has been shown that those who take first vaccine within 3 days after birth had a far better outcome (eight times better) than those who took the first dose after 7 days [9].

It has been shown that passive-active post exposure prophylaxis with hepatitis B Immunoglobulin and hepatitis B vaccine is 85 to 95% effective in preventing vertical transmission compared to 70 to 95% of vaccine alone [10]. Up to 13% of infants born to infected mothers are positive for hepatitis B infection and this is more common among children born to mothers with high viral load [10]. It has been shown that mothers who are HBV positive have a higher rate of having infants who are HBV positive and their children have a higher rate of failure to respond to hepatitis B vaccines (HBVc) [11]. Several interventions have been shown to reduce the incidence of vertical transmission. These include the use of antiviral such as tenofovir at the third trimester - if viral load is more than 1 × 10^7^ copies/ml- and vaccinating the child with both immunoglobulin and antigen soon after birth but within 24 h of birth [12]. In the Gambia such interventions are not done because there is no routine screening of pregnant women for hepatitis B infection. Infant of positive mothers only get to have the routine dose of hepatitis B vaccine usually a week after delivery, which might not be adequate for babies already exposed. Babies born to infected mothers need these interventions hence the need for a study to show the burden of the problem.

This will help policy makers to effectively plan to reduce the prevalence of hepatitis B in The Gambia. This data will also help programme managers and health planners, to plan vaccination and other preventive measures or strategies [13]. It will also show the importance of hepatitis B S antigen screening of pregnant mothers as another intervention towards the reduction of hepatitis B transmission.

Many countries have been screening pregnant women for Hepatitis B infection and these programs have helped reduced the prevalence of the disease [14]. In The Gambia, vaccination of neonates has been introduced country wide since 1990. Women who were born in the year 1990 and after are very likely to have had the vaccination against hepatitis B infection.

Hepatitis B virus (HBV) is a hepadnavirus [15]. ,Hepatitis B virus produces 9 different subtypes and genotypes (A – I) with a putative 10th genotype ‘J’ isolated from a single individual [16]. Sub-type E is commoner in The Gambia and individuals infected with genotype E have high viral loads, high frequency of Hepatitis B e antigen (HBeAg) -positivity and transmit HBV perinatally [17, 18].

Hepatitis B e antigen (HBeAg), provides a convenient, readily detectable, qualitative marker of HBV replication and relative infectivity. HBeAg positivity is associated with increased perinatal transmission [17].

The virus is transmitted by parenteral route, sexual contact and vertical transmission [19, 20]. About 10% of transmission occurs in utero [19]. Most of the vertical transmission occurs intrapartum [19]. Mothers who are both positive for Hepatitis B s antigen (HBsAg) and HBeAg have up to 90% chance of transmitting the disease to their neonates [19, 20]. Neonatal transmission mainly occurs at, or around the time of birth through the mixing of maternal blood and genital secretions. Approximately 25% of the carrier neonate will die from cirrhosis or hepatic carcinoma between late childhood and adulthood [19, 20]. Hepatitis B virus is not teratogenic [19, 20].

Pregnancy does not alter the natural history of hepatitis B infection. About 90% of pregnant women will clear the infection just like other adults [21]. Vertical transmission is the commonest mode of transmission worldwide [21]. Without immunoprophylaxis vertical transmission occurs 70 to 90% if the mothers are HBeAg positive [21]. This is reduced to 40% if the mother is HBeAg negative [21]. Overall, vertical transmission is reduced to 5 to 10% with passive and active vaccination if the mother is HBeAg positive [21]. However, mothers with high viral load despite immunoprophylaxis will have 8 to 32% of infants developing the infection and if treatment is given at the third trimester it may reduce the infection rate to almost 0 % [21]. About 1% of pregnant women will develop fulminant hepatitis, resulting in massive hepatic necrosis [21]. About 10% of pregnant women who are positive for HBsAg will become chronic carriers, and 10% of these chronic carriers will develop cirrhosis and hepatocellular carcinoma.

The drugs that can be safely used in the treatment of hepatitis B infection in pregnancy are limited due to toxicity and teratogenicity [21]. The ones found to be safe are Lamivudine, telbivudine and tenofovir [21]. Telbivudine and tenofovir in pregnancy are classified as category B by the United States Food and Drug Administration, while lamivudine is classified as C [21]. There is increased risk of developing viral drug resistance mutation with lamivudine therapy [22] Tenofovir unlike Lamivudine or telbivudine is safe during breast feeding because there are low concentrations in breast milk as it is a pro-drug [21].

Women with high viral load (more than 2000 copies per ml) should be given tenofovir in the third trimester until 3 weeks post-delivery [23]. Tenofovir is given from 32 weeks of pregnancy till 3 weeks post-delivery at a dose of 300 mg per day [23].

The aim of the study was to determine the sero-prevalence of hepatitis B virus among pregnant women attending the antenatal clinic at the Edward Francis Small Teaching Hospital, Banjul, The Gambia.

## Methods

### Study location

The study was conducted at the Edward Francis Small Teaching Hospital at the maternity department. This is the only teaching hospital in The Gambia with a 600-bed capacity. It is situated in the capital city Banjul. It is the major referral centre in the country and receives patients from the length and breadth of the country. The maternity department has a bed capacity of 90 with 3 units each headed by a consultant. Antenatal clinics are held every day. The antenatal clients seen are normal antenatal cases as well as high risk obstetric patients.

Every pregnant woman booking or continuing for antenatal care at the study location was counseled for Hepatitis B virus testing, during the study period. The pre-testing counseling was done in groups, while the post-testing counseling was done individually. Pregnant women were evaluated using history, examination, and blood test for serum HBsAg using immunographic rapid point of care test.

### Study design

This was a cross sectional study to determine the prevalence of the HBsAg among pregnant women attending antenatal clinic at the Edward Francis Small Teaching Hospital, Banjul, The Gambia. The pregnant women registering for ANC were randomly selected usually registering the 3rd whenever consent was given. This method employed a systematic sampling method. It was done to help eliminate bias.

### Study population

The study population was pregnant women attending antenatal clinic in the teaching hospital. All mothers who came for antenatal booking were counseled for hepatitis B screening in addition to the routine screening. Those who consented to be a part of the study were recruited into the study.

### Study duration

The study was conducted from 1st May to 31st July 2015. Participants were recruited from the outpatient department and the Poly Clinic of the hospital.

### Inclusion criteria

All pregnant women who came for antenatal care were told about the study and those randomly selected were recruited if they consented to be a part of the study.

### Exclusion criteria

- Any pregnant woman who declined to be part of the study..
- Any woman already in labour
- Any woman who is very ill or admitted in the ICU
- Women who are not pregnant

### Sample size determination

Earlier studies in West Africa - the geo-political zone of the study location- found that the sero-prevalence of Hepatitis B virus infection in pregnancy is 6.67% [24]. The prevalence of the disease among the sub Saharan population is shown to go up to 40%. Therefore; the minimum sample size for simple proportion with 5% accuracy, and 95% level of confidence is calculated as 368.7936. Given attrition, or none-response rate of 10%. This was rounded up to 410.

### Subjects and method

Awareness about this study was created among the staff at the antenatal clinic, polyclinic and labour ward of the Teaching Hospital during a series of focused health group discussions. Hepatitis B virus positive pregnant women were recruited into this study at the booking and antenatal follow-up clinics. Every eligible pregnant woman was counseled on the objectives of the study, and consent form was administered after ensuring that the participant fully understood the concept of the research. The mothers were tested after a needle prick on one of their fingers at the counseling room. Their results were discussed with them there before they leave. They are informed of what they need to have when they come in labour and whom to ask for in case they are not given their birth dose vaccination. Confidentiality was maintained by assigning consent enrolment code to each participant. Structured interviews were conducted at first contact with the pregnant women to collect information on demographic characteristics.

Obstetric history of each participant and gestational age was based on best available evidence. The woman’s re-collection of her date of menstrual period (L.M.P.), or earliest ultrasound scan (USS) estimate was used. Two trained midwives help obtained anthropometric measurements and the administering the questionnaires.

### Laboratory methods

The routine laboratory investigations were requested for all the patients and in addition a finger prick is done to get two drops of capillary blood to check HBsAg status using immunographic rapid test.

### Laboratory analysis

The sample was analyzed using Determine™. Determine is a point of care test that has been validated in a study in The Gambia to show high specificity and sensitivity of 100 and 85.5% respectively [25].

All analyses were done using Statistical Program for Social Sciences (SPSS). Univariate analyses were done. Chi square at significant level of 0.05 and confidence level of 95% were used to determined significance.

## Results

### Demographic characteristics

A total of 426 patients were interviewed. The youngest participant was 16 years old and the oldest 43 years old. The mean age was 28.05 years (SD5.71). Forty two percent of the participants were age between 16 and 26 years while 58% were in the age category 27 to 43 years. (Table 1). The universal vaccination for hepatitis B infection started in the country about 26 years prior to this study.

**Table 1:** Age Distribution (*N* = 424*)

Age Group	≤21	22–26	27–31	32–36	≥37	Total	2 missing data
Frequency	59	119	131	81	34	424	
%	13.9	28.1	30.9	19.1	8.0	100	

Mean age was 28.05(SD 5.7) yrs

Range 27 yrs. (16 to 43)

Of 426 patients interviewed, they listed 17 tribes to which they identified with (. Mandinka was the most frequent (38%), followed by Fula (19.5%) and then Wollof (13.1%) and then followed by Jola (7.0%).

The distributions of parity and educational level are shown in Tables 2 and 3 respectively. Most of the women (54.5%) were either nulliparous or primiparous. The gravidity followed the similar pattern. The percentage of mother that never had a miscarriage was 74.4.

**Table 2:** Distribution of parity *N* = 425

Parity	Frequency	Percentage	
Nulliparous (0)	121	28.5	There was 1 missing data.
Primiparous (1)	111	26.1	
Multiparous (2–5)	167	39.3	
Grand multiparous (≥6)	26	6.1	
Total	425	100

**Table 3:** Educational status

Educational Level	None	Primary	Secondary	Tertiary	Total
Frequency (%)	108 (26.1)	38 (9.2)	174 (42)	94 (22.7)	414^a^ (100.0)

^a^12 non-responses

Of the 426 patients, 389 (91.3%) were married, 19 (4.5%) were single and 1 (0.2%) was divorced or separated. There were 17 (4%) who did not answer on their marital status.

#### Retroviral status

Only 3 (0.7%) of the participants were seropositive for retroviral infection. Only one person had concurrent HIV infection and hepatitis B infection. It was difficult to test for a significant relationship between HIV infection and hepatitis B infection with only 3 individuals positive for HIV infection.

#### Hepatits B status

Of the 424 patients who were tested for HBsAg, 39 (9.2%) screened positive for hepatitis B. The prevalence of hepatitis B in the study population was thus 9.2%. Among the group that was likely to have been vaccinated the prevalence was 2.3% but was 13.7% among the group that was unlikely to be vaccinated.

#### Patients previously diagnosed with hepatitis B infection

Six study participants (0.6%) had previously tested positive for hepatitis prior to being tested in this study. Five of them were still positive and one was negative for hepatitis B infection. Thirty-five of those who were positive for HBsAg had never tested for hepatitis B infection. They were not aware of their condition. There was significant relationship between previously testing positive for hepatitis B infection and being positive for the infection (*P* value less than 0.001).

#### Previous relevant history

Of the 318, 8 women or study participants (1.9%) had a previous history of jaundice and 18 (4.2%) had history of blood transfusion. Seventy-six (17.9%) had history of intravenous therapy. Twenty-four (5.6%) had previous history of drug reaction.

Of the 423 participants who answered the question about tribal marks and piercing, 178 (42.1%) had either of them but majority mentioned piercing of gum to darken for beautification.

Of the respondents 62.9% (264 of the 420) said they have undergone female genital cutting.

#### Blood pressure and ANAEMIA

Of the 408 that had their urine tested 7 (1.7%) had glycosuria and 60 (14.7%) had proteinuria. 9.2% had haemoglobulin level less than 10.0 g/dl (anaemia). Of the 421 who had their blood pressure measured,19 (4.5%) had high blood pressure recordings and the remaining were normal recordings.

Tables 4, 5 and 6, have shown proportions and significant differences in hepatitis B seropositivity between likelihood of vaccinations, occupation and different age categories respectively. The prevalence rate increases with increasing age. There is strong evidence to support this relationship. There was also strong evidence to support the fact that there was a significant relationship between vaccination status and the prevalence of hepatitis B seropositivity. *P*-value less than 0.001.

**Table 4:** Vaccination status

Vaccination status	Freq	Percent	
Likely	175	41.3	2 people did not have their ages stated
Unlikely	249	58.7	
Total	424	100

**Table 5:** Occupation of the participants

Occupation	Frequency	Percent
Civil servant	*76*	*19.3*
Trader	*65*	*16.5*
Farmer	*22*	*5.6*
Unemployed	*220*	*56*
Student	*10*	*2.5*
Total	*393*	*100*

There was 33 missing data on occupation

**Table 6:** Associations between socio-demographic variables and hepatitis b seropositivity

Variable	Hepatitis Status		Total	X^2^	*P* - value (Fisher’s Exact Test)
	Sero Positive	Sero Negative			
Age (years)					
21 and less	0	58		21.958	Less than 0.001
22 to 26	4	115			
27 to 31	16	115	422		
32 to 36	10	70			
37 and older	8	26			
Marital Status					
Married	33	354	387	186	0.911
Single/separated	2	18	20		
Vaccination					
Likely	4	170		16.249	less than .001
Unlikely	34	214			
Occupation					
Civil servant	9	67		10.242	0.037
Trader	11	54			
Farmer	2	20			
Unemployed	12	206			
Student	0	10			
Miscarriage					
0	28	287		13.506	0.036
1	5	70			
2	3	14			
3	2	9			
4	0	3			
5	1	0			
Sexual Partner(s)					
1	23	280	4.936		0.026
> 1	7	32			
Drug Reaction					
yes	5	19	4.077		0.043
no	3	364			
Anaemia Status					
Anaemia	6	18	6.823		0.009
No anaemia	28	297			

There was no significant relationship between married and single patients; between nulliparous and patients who have children; between patients who work in the formal sector and those who do not, but it was shown that traders are at a significantly higher risk of developing hepatitis infection than with other occupations.

Those with more than one lifetime partners were found to be at a significantly higher risk than those with one lifetime partner. It was found that the higher the number of miscarriages the higher the chances of developing hepatitis B infection (*p* value 0.036).

The study also showed that being HBsAg positive was associated with anaemia.

There was a strong relationship between hepatitis B seropositivity and previous hepatitis B infection and history of a drug reaction (p value 0.043).

No association (even though not shown in the table) was found between the rest of the selected socio-demographic factors and hepatitis B seropositivity. Blood pressure measurement had no significant relationship. However, the very low number of seropositive might be too small to establish any statistical difference in the selected maternal characteristics. There was no significant relationship on history of blood transfusion, history of jaundice, history of intravenous drug therapy, history of tribal marks and history of FGM with hepatitis B seropositivity.

## Discussion

The primary objective of the study was to determine the hepatitis B prevalence rate in pregnant women attending the EFSTH antenatal clinics (ANC) in 2015 and to evaluate the endemicity of hepatitis B infection among pregnant women in EFSTH, a rate of less than 2% is considered low endemicity and a rate of 8% or higher is considered of high endemicity; and also, to determine if selected socio-demographic characteristics are associated with hepatitis B infection.

Thirty-nine patients were seropositive for hepatitis B infection by the point of care test which gives a prevalence of 9.2%. This prevalence rate is less than the national prevalence rate of 15% among the general population. Recent unpublished national survey by the Gambia Hepatitis Intervention Study (GHIS) has put the national prevalence at 10%. Pregnant women were excluded from this study. There is paucity of data on the prevalence of hepatitis B infection amongst pregnant women in the Gambia. The prevalence rate compares well with the study in Mali which showed also 8.0% [26]. The Mali study was also done in a tertiary hospital and among pregnant women as well. These two studies have also both found a lower prevalence among pregnant women than the general population.

It is surprising that the Gambia has a similar or higher prevalence rate than countries that it started universal HBV vaccination before. This may be due to lack of antenatal screening in the country. The high-risk neonates are not identified early and the passive immunoprophylaxis is not given. In Mali there is routine testing of pregnant women and the birth dose of the vaccine is usually given. The prevalence rate in this study is less than that of a study in Nigeria which was 11% [20]. Burkina Faso registered a very high prevalence rate of 17.3% [27]. Our prevalence rate is also far higher than some other East African countries such as Sudan (5.6%) [28], Uganda (4.9%) and Rwanda (2.4%) [29].

It is understandable to note that the prevalence among the likely to be vaccinated group was 2.3% compared to 13.7% among the unlikely to be vaccinated. This might be mainly due to the vaccination during the neonatal period. The vaccine is known to be very protective of chronic hepatitis B infection (95%) but not so well with acute infection (84%) [11, 30] It is likely that most of the infections seen in the vaccinated group are actually not chronic. It is also known that although the vaccination program became universal in the Gambia in 1990, it took about 7 years to achieve an uptake of more than 90% [5], so the women born within that first 7 years although all should have been vaccinated but this was less likely and may explain the difference in protection. Women born in the first 7 years may not be as protected as those born after the first 7 years when vaccination has been totally integrated into the EPI. This is further buttressed by the fact that the four positives in the likely to be vaccinated group were within the first 4 years of start of the universal vaccination. One was 26 years, 2 were 25 years and one was 24 years old. The older age group may also not benefit much from the protection of herd immunity as the younger group. This could explain why there was no infection among those younger than 21 years and the rate of infection continued to increase with age. It is likely that vaccination could bring down the prevalence to moderate endemicity (2 to 7%). Our study showed similar pattern to a study done in the Gambia among those born after 1990 (Vaccinated group) although among vaccinated children [5]. That study showed HBsAg prevalence of 0.5% but when it was further stratified into 5-year intervals, the prevalence of those less than 5 years became 0.2% and those between 15 and 17 years became 1.8% [5]. This trend seems to have continued in our study as shown by a prevalence of 2.5% in those between 17 and 26 years who are most likely to have been vaccinated. As the age of the vaccinated group increases so the prevalence also increases. This is explained by the waning immunity.

If only those more than 37 years are considered, the prevalence rate increases to 23.5%. This group is far from any intervention and thus has a higher prevalence rate than even the adult population of 15%. This group also is more likely to have higher rates of chronic infection.

The unvaccinated group needs to be targeted for intervention if there should be rapid decrease of hepatitis B prevalence in our general population. At present there is no intervention among this group in The Gambia. There is no screening at the ANC and birth-dose of the vaccine is hardly ever given. The options we have are to either wait until the youngest age category of the unvaccinated group reaches menopause which is in 24 years’ time or to start intervention soonest in order to see a drop in the prevalence of the disease. Mali is intervening at this level by the screening of pregnant women and ensuring that the birth dose is given and this might explain why the prevalence rate in our study is higher than in Mali despite The Gambia starting the universal vaccination program before Mali.

The Gambia has been very interested in the fight against hepatitis B infection and was among the first countries in the world to initiate a universal program for neonatal vaccination. It is surprising to see the fight has only been directed towards neonatal vaccination where successes have been continuously registered for more than 25 years now. The lack of intervention among pregnant women has been fueled by few studies showing vertical transmission is less of a problem compared to horizontal transmission among children [5, 14]. However, majority of studies show that in reality, it is otherwise [31]. Our study was consistent with other studies that showed hepatitis B sero-positivity did not have any relationship with history of jaundice or female genital cutting (FGC) [32, 33]. The prevalence of the FGC in this study is similar to the national prevalence of 79% [34].

Anaemia was found to be significantly related to hepatitis B sero-positivity (*P* value 0.009). The reason is not so clear and need to be further studied. It maybe that hepatitis infection may affect the production of proteins that carry iron for erythrocytosis. Miscarriage was strongly related to Hepatitis B sero-positivity (*P* value 0.036). This could be as a result of exposure to unsterile instruments during procedures to evacuate the uterus after a miscarriage. A study suggested that the increase in abortion rate among hepatitis B infected mothers was mainly due to infection of embryos [35]. Those with history of Hepatitis B infection in the past were predominantly positive again. Only one of the six who previously tested positive was negative. This shows that these are most likely to be chronic sero-positivity but a recall bias may also be a factor here.

Those who work as traders were also more likely to get the hepatitis B infection. This is not surprising as in The Gambia, traders are a vulnerable group as they are more likely to have multiple sexual partners. Most of the HIV hot spots in the Gambia are in “lumoos” (these are local trading points usually in villages). This is quite different from what was obtained in a study in Nigeria where traders were less at the risk than the students or civil servants [36].

From the results, no significant statistical difference was established between the other selected sociodemographic factors and hepatitis B seropositivity.

## Limitations

### Limitations of the study

1. The small number of seropositive hepatitis B patients made it difficult to establish more associations between socio-demographic factors and hepatitis sero-positivity. A larger sample size would have been better.
2. Inability to check for Hepatitis E antigen and viral load was a big limitation as it could have pointed to the most likely efficient type of intervention needed to prevent vertical transmission i.e. whether anti-viral treatment or immunization or a combination of both.
3. Inability to analyse for chronicity was also a limitation as it would have allowed further analysis of the prevalence of chronicity. This is important as the vaccine given at the neonatal period is known to protect against chronic disease very well but not so well with acute hepatitis B infection.
4. The inability to precisely determine those who received vaccination and those who did not might bring in some recall bias.

## Conclusion

The prevalence rate of hepatitis B sero-positivity among pregnant women attending the antenatal clinic of Edward Francis Small Teaching Hospital (EFSTH) is 9.2%. The prevalence rate amongst the likely vaccinated cohort is 2.3% and the rate among the unlikely to have been vaccinated cohort is 13.7%.

Likelihood of vaccination was significantly related to HBV sero-positivity.

## Abbreviations

ALT: Alanine transaminase
ANC: Antenatal clinic
DNA: Deoxyribo Nucleic Acid
e: Envelope
EASL: European Association for the Study of the Liver
EFSTH: Edward Francis Small Teaching Hospital
Elisa: Enzyme-link immunosorbent assay
EPI: Program of immunisation
FGC: Female Genital cutting
FGM: Female Genital Mutilation
GHIS: Gambia Hepatitis Intervention Study
HB _e_ Ag: Hepatitis B Virus E antigen
HBcAg: Hepatitis B core antigen
HBeAg: Envelope antigen
HBsAg: Hepatitis B Surface antigen
HBV: Hepatitis B Virus
HCC: Hepato-Cellular Carcinoma
IU: International unit
L: Liters
L.M.P: Last menstrual period
ml: millilitre
UNICEF: United Nations Children’s Fund
USS: Ultrasound scan
WHO: World Health Organisation
